# Effects of psilocybin microdosing on awe and aesthetic experiences: a preregistered field and lab-based study

**DOI:** 10.1007/s00213-021-05857-0

**Published:** 2021-04-30

**Authors:** Michiel van Elk, George Fejer, Pascal Lempe, Luisa Prochazckova, Martin Kuchar, Katerina Hajkova, Josephine Marschall

**Affiliations:** 1 Institute of Psychology, Leiden University, Wassenaarseweg 52, 2333 AK Leiden, The Netherlands; 2 Department of Psychology, University of Amsterdam, Nieuwe Achtergracht 129B, 1018WT Amsterdam, The Netherlands; 3 Forensic Laboratory of Biologically Active Substances, Department of Chemistry of Natural Compounds, University of Chemistry and Technology Prague, Prague, Czech Republic; 4 Department of Experimental Neurobiology, National Institute of Mental Health, Klecany, Czech Republic

**Keywords:** Psilocybin, Microdosing, Awe, Aesthetics, Expectancy-effects

## Abstract

There is an increased societal trend to engage in microdosing, in which small sub-hallucinogenic amounts of psychedelics are consumed on a regular basis. Following subjective reports that microdosing enhances the experience of nature and art, in the present study we set out to study the effects of psilocybin microdosing on feelings of awe and art perception. In this preregistered combined field- and lab-based study, participants took part in a microdosing workshop after which they volunteered to self-administer a psilocybin microdose or a placebo for three consecutive weeks, while the condition was kept blind to the participants and researchers. Following a 2-week break, the condition assignment was reversed. During each block, participants visited the lab twice to measure the effects of psilocybin microdosing vs. placebo. We used standardized measures of awe, in which participants reported their experiences in response to short videos or when viewing abstract artworks from different painters. Our confirmatory analyses showed that participants felt more awe in response to videos representing funny animals and moving objects in the microdosing compared to the placebo condition. However, about two-third of our participants were breaking blind to their experimental condition. Our exploratory findings suggest that expectancy-effects may be a driving factor underlying the subjective benefits of microdosing.

**Supplementary Information:**

The online version contains supplementary material available at 10.1007/s00213-021-05857-0.

## Introduction

Serotonergic hallucinogens—which can be considered a subclass of the broader category of psychedelics—include mind-altering substances, such as LSD and psilocybin. In our society there is an increasing trend for the recreational use of hallucinogens, as evidenced by the National and European Drug Monitor (European Monitoring Centre for Drugs and Drug Addiction (EMCDDA) 2019; Van Laar and Gestel 2017) and the Global Drug Survey (GDS), indicating for instance that 40% of the people who ever used LSD started using it in the past year (Winstock et al. 2018). More than 70 centers in the Netherlands currently offer recreational ayahuasca or psilocybin retreats, which typically last several days and are organized in a ritual setting. We are currently also witnessing an increased interest in scientific research on hallucinogens: psychedelics are increasingly used for clinical purposes and in neurocognitive studies (Carhart-Harris et al. 2014, 2016; Griffiths et al. 2006). Psychedelics are claimed to have a strong therapeutic potential for the treatment of biomedical disorders, including severe depression, substance abuse, and cancer-related anxiety disorders (Bogenschutz et al. 2015; Krebs and Johansen 2012; Kyzar et al. 2017; Rucker et al. 2018).

Of interest for the present project is the recent hype to engage in LSD or psilocybin microdosing (e.g., the prevalence of LSD microdosing among GDS respondents was 28.6%), in which small amounts of hallucinogens are consumed on a regular basis. People microdose for many different reasons, ranging from experiencing increased flow and creativity to relief from cluster headaches to dealing with anxiety and depression (Anderson et al. 2019). As of yet the alleged beneficial effects of microdosing mainly rely on anecdotal reports on websites and forums such as Reddit and Erowid—reinforcing the idea that microdosing is a hype among young professionals and high potentials working in the tech industry. However, systematic research on the topic is scarce and there are currently no scientifically informed guidelines or best practices surrounding the use of microdosing (Kuypers et al. 2019).

The few existing studies on microdosing provide mixed evidence regarding its efficacy. By using self-report measures it has been found that microdosing positively affected mood, creativity, and cognition, while reducing anxiety and depression (Johnstad 2018). People who microdosed experienced more wisdom, open-mindedness, and creativity (Anderson et al. 2019; Petranker et al. 2020a, b). In a large-scale study it was found that microdosing enhanced mood and overall well-being (Polito and Stevenson 2019). The self-reported effectiveness of microdosing for mental problems was higher than other (conventional) methods to treat attention- and anxiety-related disorder, although the effects were smaller than those experienced following a full psychedelic dose (Hutten et al. 2019). Next to its positive effects on reducing depression and stress, microdosing increased the tendency to become absorbed in external stimuli and also induced an increase in the personality trait of neuroticism (Polito and Stevenson 2019). However, most of these studies rely entirely on (retrospective) self-report measures, there was a strong selection bias in the sample, and there was no control condition. It is thus unclear to what extent the observed outcomes are driven by expectancy-effects, demand characteristics, and socially desirable responding.

Experimental research on microdosing has shown that psilocybin and ketamine microdosing in rats did not induce clear anxiolytic effects (Horsley et al. 2018). In another study chronic microdosing with DMT did have antidepressant-like effects in rats, as evidenced by their performance on a forced swim test (Cameron et al. 2019). In humans an acute (non-blinded) dose of psilocybin increased convergent and divergent thinking (Prochazkova et al. 2018). However, this study was not placebo-controlled, which might have increased expectancy-effects, the dosage used was rather low, and next to the effects on creativity other measures were included for which microdosing did not appear to have an effect. In placebo-controlled studies it has been found that LSD microdosing (i.e., the effects were most pronounced at 10 µg of LSD) dilates time-perception as measured using a temporal reproduction task (Yanakieva et al. 2019). LSD microdosing (i.e., at the highest dosage of 26 µg of LSD, which can no longer be considered as sub-hallucinogenic) also decreased the positivity ratings of images with positive content (Bershad et al. 2019). A recent fMRI study found that a microdose of LSD (i.e., 13 µg of tartrate LSD) compared to a placebo condition increased functional connectivity between the amygdala and the middle-frontal gyrus, which were in turn related to changes in positive mood (Bershad et al. 2020). Another study found no impairments of different microdoses of LSD (5, 10 and 20 µg of tartrate LSD) on different measures of cognitive performance, such as spatial and working memory, visual attention, balance, and proprioception (Family et al. 2020). Finally, a recent study found that LSD microdosing (5, 10, and 20 µg of base LSD) differentially affected a wide range of variables, including sustained attention, speed of information processing, mood states, anxiety, and confusion (Hutten et al. 2020). Thus, experimental studies indicate that microdoses can indeed affect implicit cognitive processing—albeit sometimes in an unexpected direction.

The lack of a clear and consistent effect of microdosing on subjective and objective performance might be related to differences in the methodology that was used. For instance, differences in the preparation of the microdose (tartrate vs. base LSD) may make the outcomes between studies difficult to compare (Holze et al. 2021). Furthermore, in many studies the ecological validity of the dependent cognitive measures was low, as the highly standardized and controlled experimental paradigms and scales do not fit well with the complex and multifaceted subjective nature of the psychedelic and microdosing experience. For instance, visual attention tasks typically involve the repeated presentation of trials to which the participant is required to respond, making performance on these tasks highly contingent on intrinsic motivation and familiarity (e.g., specifically university students are often over-trained on these experimental paradigms, while naïve participants may find the same tasks often more challenging). In order to move the field forward we need to infuse the study of microdosing psychedelics with more ecologically valid manipulations and measures. Here we describe the results of a pre-registered placebo-controlled study in which we had the unique opportunity to study the effects of psilocybin microdosing on feelings of awe and aesthetic experiences.

Awe is a complex emotion that is typically elicited by perceptually vast stimuli such as landscapes, vistas, and mountains (Shiota et al. 2007). It is characterized by perceived vastness and a need for accommodation, resulting in the need to revise one’s existing mental models (Keltner and Haidt 2003). Recently it has been suggested that the alleged therapeutic potential of psychedelics relies on its awe-inducing properties (Hendricks 2018; Johnson et al. 2019): during a psychedelic experience the user is confronted with profound insights, visionary experiences, the experience of ego-loss, and personal transgression. These transformative experiences in turn have a positive effect on subsequent well-being, feelings of self-compassion, and connectedness to others (Forstmann et al. 2020).

Preliminary evidence for the hypothesized relationship between psychedelics and awe can be found in research on the personality trait of absorption (Lifshitz et al. 2019). The Tellegen absorption scale was originally developed to measure hypnosis-proneness (Tellegen and Atkinson 1974) but in subsequent research turned out to be an interesting personality factor that captures a proclivity for vivid mental imagery, openness to immersive sensory experiences and to get absorbed in one’s inner mental life. Previous studies have shown that the personality trait of absorption predicts responsiveness to psychedelics (Studerus et al. 2012), as well as feelings of awe in response to natural scenes (van Elk et al. 2016). At a neural level, it has been found that awe-experiences in response to vast natural scenes are characterized by a decreased activity of the default mode network (DMN)—a network of brain regions involved in mind-wandering and self-referential processing (van Elk et al. 2019). More specifically, when healthy participants were immersed in awe-inducing videos of nature, the DMN was less active compared to when the participants were observing funny videos or neutral videos. Interestingly, a similar decrease in DMN activity has been found in association with the acute effects of LSD and psilocybin on resting state network activity (Carhart-Harris et al. 2012, 2016). Both feelings of awe and psychedelic experiences could thus share a similar underlying neurocognitive mechanism, related to a reduced focus on the self and an immersion in the sensory nature of the experience. We note that as-of-yet we do not know whether psilocybin microdosing results in a similar change of DMN activity as has been observed for a full psychedelic experience. Still, based on these convergent lines of evidence, in the present study we hypothesized that psilocybin microdosing would increase feelings of awe in response to the same videos that we used in previous research (van Elk et al. 2019).

It has also been suggested that psychedelics can enhance creativity and art production and perception (Spee et al. 2018). Anecdotal reports (e.g., at Reddit) suggest that microdosing increases the enjoyment of the arts and museum visits as well: artworks are perceived to be more vibrant, to have more “depth,” and to have more meaning to the spectator. Absorption has been found to predict the intensity of aesthetic experiences as well (Silvia and Nusbaum 2011; Wild et al. 1995) and aesthetic experiences have been associated with an increased activation of the DMN, which likely reflects that participants during aesthetic judgments relate the observed artworks to themselves (Belfi et al. 2019; Vessel et al. 2012). Thus, in the present study we hypothesized that psilocybin microdosing would increase the perceived profoundness of abstract works of art.

In order to study the effects of psilocybin microdosing in a systematic and controlled fashion, we used the opportunity to set up a combined field- and lab-based study, in which participants self-administered a psilocybin microdose or a placebo. One group of participants started off with psilocybin microdosing for 3 weeks, while the other group received a placebo. In these weeks the participants visited the lab at the University of Amsterdam twice, during which they were administered our awe and art perception tasks. Following a 2-week break, the condition assignment was reversed, such that the psilocybin first group now self-administered a placebo and vice versa for the placebo first group. The condition assignment was kept blind from both the participants and the experimenters and the blinding information was only revealed after the data collection and analysis had been finalized. Next to the awe and art perception studies reported in this manuscript, we also included other cognitive and behavioral measures to assess the effects of psilocybin microdosing on, e.g., temporal recalibration, emotion perception, creativity, and bistable perception. The full package of the studies and the hypotheses can be found online (https://osf.io/cn8z4/) and we will report the results from the other studies elsewhere. The awe and art perception task were administered towards the end of the testing block, thus about 1 h after participants were in the lab. In this manuscript we have reported all measures, conditions, and data exclusions. We did not run a power analysis prior to the study as we did not know which effect-size to expect for our different cognitive tasks and because we were dependent on the availability and willingness of participants who volunteered to participate in the microdosing workshop (for details, see below).

Our awe manipulation was directly based upon the stimulus material we used in previous studies (van Elk et al. 2016) and consisted of three types of videos: awe-inducing videos, representing vast natural scenes; positive videos, representing funny animals; and neutral videos, representing man-made objects and boring landscapes. These videos have been extensively pre-tested on different samples and using different dependent measures in prior studies. The main rationale for including positive and neutral videos was to control for the positive valence and the high arousing nature of the awe videos: this way we could investigate whether any eventual effect of microdosing specifically affects feelings of awe. Next to measuring the effects of psilocybin microdosing on awe we also assessed participants’ implicit perception of their body. In previous studies it has been found that feelings of awe are characterized by the experience of a small self (Bai et al. 2017; Piff et al. 2015; Preston and Shin 2017). For instance, when prompted with a pictorial representation of their body, after watching an awe video participants indicated that the size of their body was smaller, compared to watching a control video (van Elk et al. 2016). In this study we aimed to extend these findings, by investigating whether psilocybin affects feelings of awe and whether awe-experiences are also accompanied by an underestimation of one’s body size.

For the art perception task we selected abstract artworks from four different artists and participants were asked to indicate the profoundness of the artworks and the positive and negative emotions that the artwork elicited.

### Hypotheses

All hypotheses, the experimental material and procedure and statistical analysis plan were pre-registered on the Open Science Framework (https://osf.io/cn8z4/). For the awe videos, we specifically set out to test the following hypotheses:

- **H1**_**awe**_ Feelings of awe will be higher (and body size estimates smaller) for Awe compared to Positive and Control videos (i.e., main effect of Video).
- **H2**_**awe**_ Feelings of awe will be higher (and body size estimates smaller) when people are microdosing compared to the placebo condition—especially in response to awe videos (i.e., we expected an interaction between Condition and Video).
- **H3**_**awe**_ We expect the effect of microdosing on awe to be stronger in the 1st compared to the 2nd session, which should be reflected in an interaction between Condition, Video, and Testing Session.
- **H4**_**awe**_ We expect the effect of microdosing on awe to be stronger when comparing participants in the first block on vs. off microdosing (i.e., as these participants were not yet “habituated” to the awe videos which are repeatedly presented and not habituated to the microdosing effects): this should be reflected in an interaction between Condition, Video, and Block Order (i.e., most pronounced effect of microdosing expected when looking at the between-subjects comparison for the first session).
- **H5**_**awe**_ In an additional analysis we will include the Tellegen absorption scale as a covariate. Based on previous studies we expect a main effect of Absorption on feelings of awe and body perception: high absorption participants will report stronger feelings of awe and perceive their body to be smaller. We also expect the effect of microdosing on felt awe to be higher for high absorption participants, which should be reflected in an interaction between Condition, Video, and Absorption.

For the art perception task, we specified the following hypotheses:

- **H1**_**art**_ Positive emotions in response to the artworks will be higher in the on vs. off microdosing condition (main effect of Condition). Negative emotions will be reduced in response to the artworks in the on vs. off microdosing condition.
- **H2**_**art**_ This effect is expected to be stronger in the 1st compared to the 2nd testing session (i.e., we expect an interaction between Condition and Testing Sessions) and is also expected to be stronger in the first compared to the second half of all testing sessions (i.e., interaction between Condition and Block Order).
- **H3**_**art**_ In an additional analysis we will include the Tellegen absorption scale as a covariate. We expect a main effect of Absorption for positive emotions in response to artworks: high absorption participants will report more positive emotions. We also expect the effect of microdosing on positive emotions to be higher for high absorption participants, which should be reflected in an interaction between Condition and Absorption.

## Methods

### Deviations from pre-registration

In our preregistered analysis plan we indicated that we would include block order in the statistical design. As indicated below, due to a programming error, the different videos and artworks were not fully counterbalanced between the different sessions and blocks. Accordingly, the interaction-effects with block order are partly confounded with different stimulus types being presented to participants, and therefore need to be interpreted with caution.

### Participants

Prior to the study participants were asked for contra-indications, including a prior diagnosis or family problems with schizophrenia, psychosis, mania, or borderline. We also excluded participants who indicated to have an addiction, who had serious physical health issues (e.g., diabetes or brain injury) and who lacked proficient English language skills (as the study was conducted in English). All participants provided their written informed consent to participate in the study. We provided participants with the guidelines as specified by the researchers involved in this project, asking them to comply with the proposed microdosing schedule for 2 months, self-administering the dose 1.5 h prior to the lab session, to remain blind to their condition, to self-administer at least five of the seven microdoses per block, and to refrain from using other psychoactive substances and medications during the study. The duration of 1.5 h after ingesting the microdosing capsule is based on the observation that the subjective effects of a higher dose of psilocybin and the plasma levels tend to peak around 90 min after intake (Passie et al. 2002).

In total 75 participants started out with our study, but 20 dropped out during the first testing block. Fifteen did not comply with the behavioral guidelines, e.g., they took the dose less than 45 min or longer than 2.5 h before the experimental session or they took other psychoactive substances in the weeks prior to the study. Of an additional 10 participants (12 for the art perception study), we were not able to collect all data points from either the four lab-based testing sessions or the two post-tests. Thus, we included 30 participants (28 for the art perception study) in our final analysis, consisting of 17 females, mean age = 29.1 years, age range = 20–48. Thirteen participants started out with the psilocybin first condition, while the other 17 participants started out with the placebo first condition. The groups did not differ in age (psilocybin first, mean age = 30.9 years; placebo first: mean age = 27.8 years). We do not have quantifiable data regarding past drug use or prior experience with psychedelics from the participants who ended up in our final analyses. However, informal observations and responses during the microdosing workshop indicated that the majority of all participants had prior experience with using psychedelics and/or with microdosing.

### Microdosing workshop and ethical approval

Study participants interested in experimenting with microdosing psychedelics were recruited through a bi-yearly “Microdosing Information Workshop” organized by the Psychedelic Society of the Netherlands (PSN). At this event, participants learned how to create their own batch of doses (7 in total) and some began taking these in the weeks following. The presentation that was presented to participants at the workshop has been uploaded on the OSF under “workshop materials” (https://osf.io/e5zdx/). The PSN offered us the opportunity to contact their guests for quantitative follow-up assessments into the effects of microdosing and to distribute placebo samples at the event so that participants could engage in self-blinding. In order to ensure honest self-report and to protect the participants’ interests, their identity was kept anonymous from the researchers via the PSN. The study was approved by the local ethics committee at the University of Amsterdam (project no. 2019-SP-10060; see: lab.uva.nl). This study entailed no deception and participants were fully informed about the purpose of the study. After the study ended, the condition information (i.e., whether they microdosed in the first or the second block) was revealed to participants.

### Study design

Prior to the study participants completed a survey including demographic questions, the Tellegen absorption scale (Tellegen and Atkinson 1974) and we asked participants about their expectations about the alleged effects of microdosing, by indicating their agreement with 20 statements (e.g., By microdosing I hope to…. “improve my mood,” “increase my focus,” “get more easily into the flow,” etc.; see Appendix for all questions; the items were completed using a 10-point scale ranging from 1 = not at all to 10 = very much). All participants attended a microdosing workshop, during which they were informed about best microdosing practices, as well as about the study setup and design.

In the subsequent 3 weeks participants engaged in a microdosing schedule (i.e., 1 day of microdosing followed by a 2-day interval), during which they took a total number of 5–7 microdoses of psilocybin consisting of 0.7 g dried psilocybin-containing truffles or a placebo, consisting of 0.7 g of regular dried mushrooms and rice/seeds to add weight to the capsule, which were orally administered in white capsules. The microdose corresponded approximately to 1.5 mg of psilocybin (see Truffle analysis below) and participants always took their microdose at home. During the 3-week period they visited the University of Amsterdam twice (i.e., in week 1 and week 3) to measure the effects of microdosing (i.e., participants took their microdose no more than 1.5 h prior to the lab session). Each lab session lasted approximately 1 h in total. After participating in the lab-based testing, participants were allowed to go home by themselves, after it was ascertained by the experimenter that they did not experience any adverse side effects as a consequence of the microdose. During the entire experimental session a PSN member was present in the lobby to provide and assist in case any adverse side effects were experienced (this never happened during the entire study).

The first block was followed by a 2-week break during which participants did not microdose. Participants completed a survey in which we asked them to guess their condition in the prior weeks and we asked them about their expectations of microdosing for the subsequent block of 3 weeks (see Appendix for the expectation questions).

Next, participants again engaged in a 3-week microdosing schedule in which they took 5–7 microdoses of psilocybin or placebo. The condition assignment was reversed such that participants who started with psilocybin in the first block, now received a placebo, while participants who started with a placebo received psilocybin in the second block. During the entire study the assignment of conditions was never revealed neither to the participants nor to the experimenters. Participants again visited our lab twice (i.e., in weeks 6 and 8) to measure the effects of the microdose.

The second block was followed by a final survey in which we asked participants again to guess their condition in the preceding 3 weeks. We detail the awe and art perception measures that we included in our study below.

As the current trial was a collaborative effort among several researchers, next to the measures reported here, other experimental paradigms with distinct theoretical background and research interests were included in the current trial: an emotional Go/No-Go task; an audio-visual integration task; a temporal reproduction task; a bistable perception task; measures to assess depression and anxiety; and measures to assess interoceptive awareness. For these different tasks, the hypotheses are specified on the OSF (https://osf.io/cn8z4/), but the findings regarding these tasks will be reported elsewhere.

### Feelings of awe

**Fig. 1:**
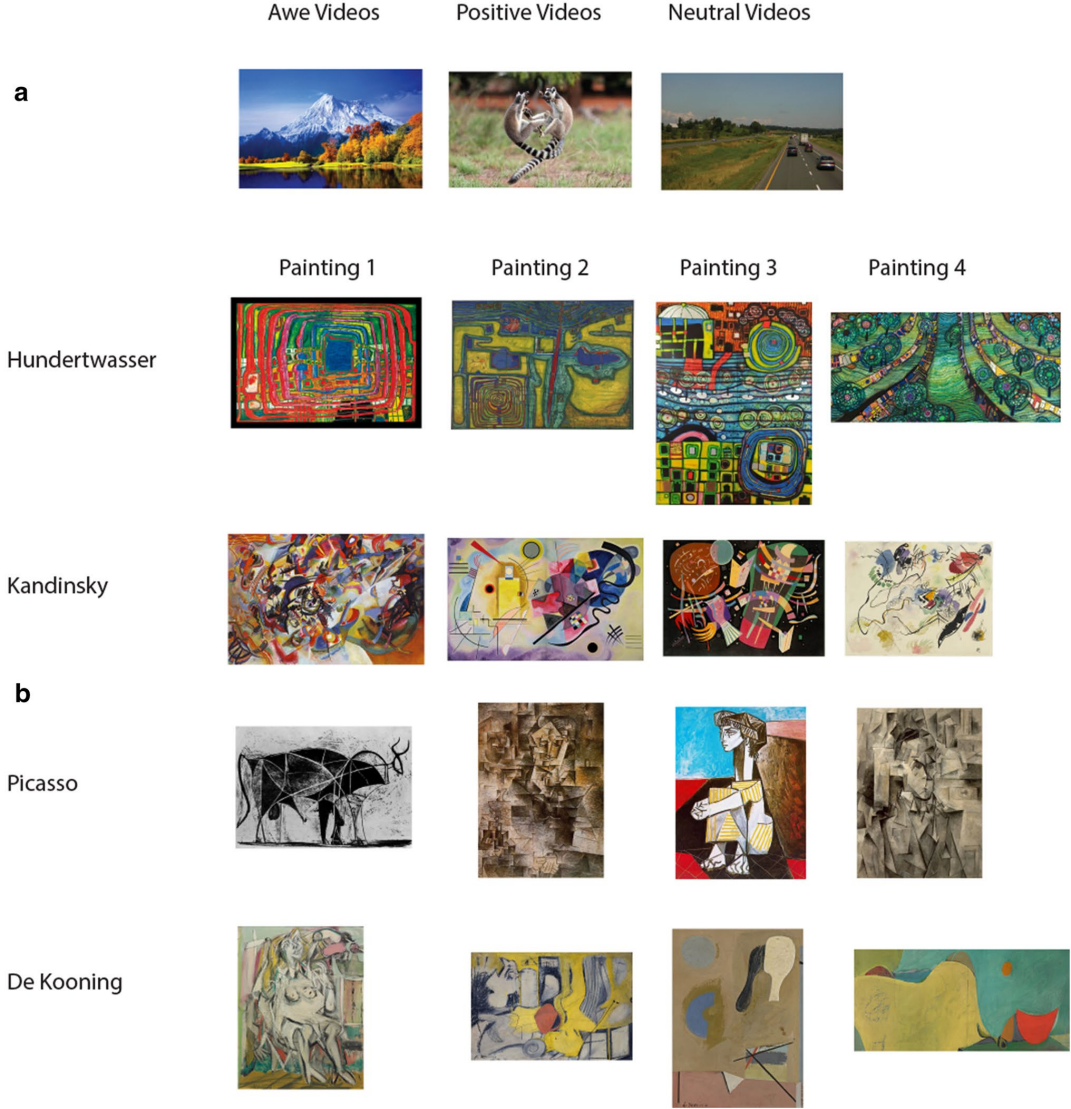
**a** In the awe task participants were presented with videos representing vast natural scenes (awe), funny animals (positive), or moving manmade objects (neutral). **b** In the art perception task participants were presented with artworks from four different painters

We measured participants’ feelings of awe during 4 consecutive lab-based testing sessions, in which participants either took a psilocybin microdose or a placebo. During each session, participants were presented with 2 awe videos, 2 positive videos, and 2 control videos (see Fig. 1). Following each video participants were required to rate their felt awe by using 5 questions (see Table 1) and they were provided with a pictorial body size estimation task (similar to the measures used in van Elk et al. 2016). The 5 questions used in the present study correspond to the following questions in the original study (van Elk et al. 2016): no. 1, no. 2, no. 3, no. 4, and no. 6. The body size estimation task consisted of a pictorial representation of the participant in the experimental room, representing the size of the body with respect to the room in different ratios. By using this measure, in our previous study we found that when participants are experiencing awe, they tend to underestimate the size of their body with respect to the experimental room (van Elk et al. 2016). In this study we aimed to replicate and extend these findings: that is, we expected that participants would further underestimate their body when watching awe videos when in the psilocybin microdosing compared to the placebo condition (H1_awe_ and H2_awe_).

**Table 1:** Questions that were used to assess feelings of awe in the present study

To what extent did watching the video induce the experience of something beautiful?
To what extent did the video induce the feeling that ultimately all life is one?
To what extent did the video induce feelings of self-transcendence?
To what extent did you experience a loss of sense of space and time during watching the video?
To what extent were you impressed by watching the video?

In total 8 awe videos, 8 positive videos, and 8 control videos were presented, which are all available on the OSF (https://osf.io/9zwqy/files/). All videos have been pretested in a previous study (van Elk et al. 2019). We made sure that participants only saw each video once, i.e., in a different session and experimental condition. Due to a programming error the different types of videos were not correctly counterbalanced over the different sessions. Accordingly, assessing the effects of block order (i.e., psilocybin vs. placebo first) was partly confounded with assessing differences in the types of videos that were presented. This confound does not compromise our main effects of interest however (i.e., main effect of Condition and Video).

### Art perception

We measured participants’ aesthetic responses to abstract artworks in 4 consecutive testing sessions, in which participants took an acute microdose of psilocybin or a placebo. During each session, participants were presented with an abstract artwork by Willem de Kooning, Friedensreich Hundertwasser, Wassily Kandinsky, and Pablo Picasso (see Fig. 1). In total 4 paintings from each artist were presented, distributed across the different testing sessions. Due to a programming error the different types of artworks were not correctly counterbalanced over the different sessions. However, in each block paintings of each of the four different painters were presented. Accordingly, assessing the effects of block order (i.e., psilocybin vs. placebo first) was partly confounded with assessing differences in the types of artworks that were presented. This confound does not compromise our main effects of interest however (i.e., main effect of Condition and Painter).

## Statistical analysis

The 5 awe ratings for each video were combined in a total awe score and we averaged the awe rating over each testing session per condition. As specified in the preregistration, for the awe ratings and the body size measure we used a repeated measures ANOVA with the within-subjects factors Condition (psilocybin vs. placebo), Video (Awe, Positive, Control), and Testing Session (1st vs. 2nd). For the critical comparison in our study, we used 4 omnibus ANOVAs, with as dependent measures (1) feelings of awe, (2) body size perception, (3) positive emotions, and (4) negative emotions. In order to correct for multiple comparisons, we lowered the statistical threshold to consider effects significant to *p* = 0.025. Post-hoc comparisons were corrected for multiple comparisons, using Bonferroni correction, by adjusting the critical *p*-value (*p* < 0.05) according to the number of comparisons that was made.

Following each artwork participants were required to indicate the perceived profoundness of the artwork and the positive and negative emotions elicited by the artwork, by using 9 items (the measures were derived from Vessel et al. 2012). The emotion ratings were combined in a positive and a negative experience score. As specified in the preregistration, for the positive and negative ratings we used repeated measures ANOVA with the within-subjects factors Condition (psilocybin vs. placebo), Artist (Kooning, Hundertwasser, Kandinsky, Picasso), and Testing Session (1st vs. 2nd). Post-hoc comparisons were corrected for multiple comparisons, using Bonferroni corrections, by adjusting the critical *p*-value (*p* < 0.05) according to the number of comparisons that was made.

### Drugs and chemicals

In our study, in the psilocybin condition participants took 0.7 g of dried psilocybin-containing truffles. In order to determine the potency of the psilocybin-containing truffles that were used by the microdosing participants, samples of the truffles were sent for analysis to the Czech Republic and were analyzed by MK and KH. The full details and outcomes of this analysis are described in the supplementary material online. The results in Supplementary Table 1 are written in micrograms per gram, so they have to be divided by 1000 to obtain mg. For each dose participants in our study consumed the equivalent of 0.7 g of truffles that were dried at 25 °C using a regular heater. Thus our participants had about 1.5 mg psilocybin per dose (although our drying method may have been less controlled than the lab-based method). Participants were instructed that they could best store their capsules in the fridge. In total, our study lasted approximately 2 months. In a previous study using a similar setup, we analyzed the content of the truffles over time and we found that the amount of psilocybin in the dried truffles remained stable (Prochazkova et al. 2018). Although there is no accepted scientific definition as of how a microdose should be defined, for practical purposes it is often considered to be one-tenth of a dose that elicits hallucinogenic effects (Kuypers 2020). A recent survey indicates that users reported taking between 0.2 and 0.5 g of dried psilocybin mushrooms (Fadiman and Korb 2019). Thus, the dosage that we used in our study (0.7 g of dried truffles) was at the high end of the spectrum, thereby potentially increasing the likelihood of finding an effect, but also causing participants to break blind regarding their condition assignment (see below).

## Results

### Confirmatory analyses

#### Effects of psilocybin on feelings of awe

**Fig. 2:**
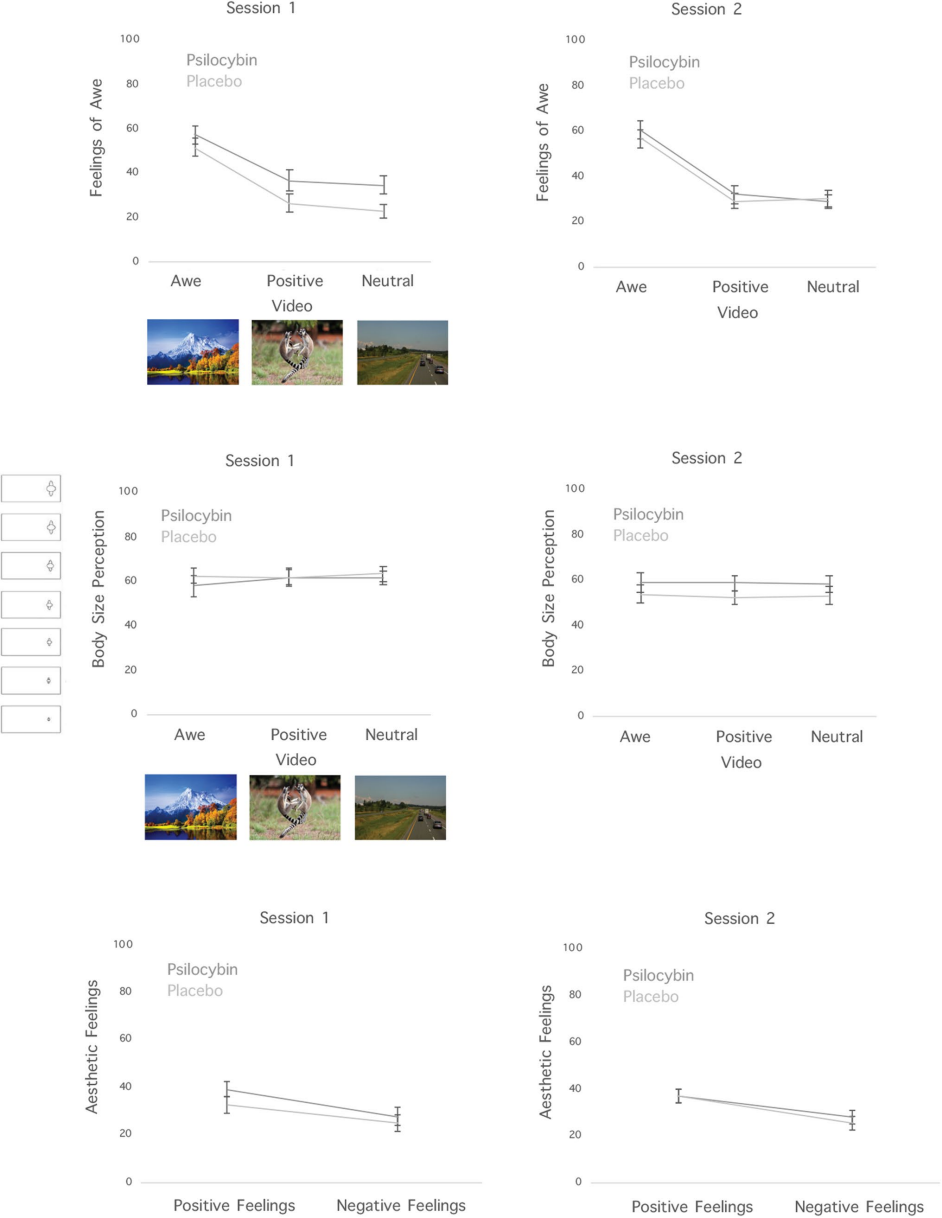
Effects of psilocybin microdosing on feelings of awe in response to awe, positive and neutral videos for the different experimental sessions and blocks (upper panel). Effects of psilocybin microdosing on body size perception for the different experimental sessions and blocks (middle panel). Effects of psilocybin microdosing on positive and negative feelings in response to abstract artworks (lower panel). Error bars represent one standard error

The descriptives for feelings of awe in the different experimental conditions are presented in Table 2. In line with our preregistered H1_awe_, we observed a main effect of Video, *F*(2, 58) = 94.144, *p* < 0.001, *η*^2^ = 0.765, indicating that our experimental manipulation was successful and that awe videos induced stronger feelings of awe than positive and control videos (see Fig. 2). Also H2_awe_ was confirmed: we found a main effect of Condition, *F*(1, 29) = 8.309, *p* = 0.007, *η*^2^ = 0.223, reflecting that participants felt more awe in the microdosing compared to the placebo condition (see Fig. 2).

**Table 2:** Feelings of awe in the different experimental conditions

Video	Condition	Session	Mean	SD
Awe	Placebo	Session1	51.493	22.311
		Session2	56.797	20.877
	Psilocybin	Session1	57.513	22.508
		Session2	60.577	20.545
Ntr	Placebo	Session1	22.693	17.667
		Session2	30.527	21.431
	Psilocybin	Session1	34.65	22.569
		Session2	29.077	16.698
Pos	Placebo	Session1	26.543	21.388
		Session2	29.457	19.562
	Psilocybin	Session1	36.563	25.522
		Session2	32.167	22.259

In line with our H3_awe_, we found a marginally interaction between Condition and Session: *F*(1, 29) = 4.934, *p* = 0.034, *η*^2^ = 0.145, which was further qualified by a three-way interaction between Condition, Session, and Video, *F*(2, 58) = 3.308, *p* = 0.044, *η*^2^ = 0.102. However, this effect did not survive correction for multiple comparisons.

To test our H4_awe_ we included block order as an additional between-subjects factor in our statistical design. Indeed, in line with our expectations we found an interaction between Block Order, Condition, and Video, *F*(2, 56) = 3.950, *p* = 0.025, *η*^2^ = 0.004. As can be seen in Fig. 2, this interaction reflected that participants felt more awe in the psilocybin condition to positive and neutral videos, but only in the first block, but not during the second block of the experiment. We also found a significant interaction between Block Order, Condition, and Session, *F*(1, 28) = 10.502, *p* = 0.003, *η*^2^ = 0.010. This interaction reflected that feelings of awe were only affected by psilocybin in the first session of the first block, whereas in the other experimental sessions, there was no effect of psilocybin microdosing on awe (see Fig. 2).

To test H5_awe_ we included absorption as a covariate in the analysis. In line with our predictions, we found a main effect of Absorption, *F*(1, 28) = 18.36, *p* < 0.001, *η*^2^ = 0.396, reflecting that high absorption participants overall reported stronger feelings of awe. However, in contrast to our second prediction, absorption did not interact with Condition (*F*(1,28) = 0.92, *p* = 0.346, *η*^2^ = 0.002).

#### Effects of psilocybin on body size estimation

The descriptives for body size estimation in the different experimental conditions are presented in Table 3. The same confirmatory hypotheses as conducted for the awe ratings were conducted for the body-perception measures. In contrast to our H1_awe_ we did not find that awe videos resulted in smaller body size estimates (*F*(2,58) = 0.118, *p* = 0.889). We also found no evidence for H2_awe_: psilocybin microdosing did not decrease body size estimates (*F*(1, 29) = 0.766, *p* = 0.389). We found partial evidence for H3_awe_: the interaction between Condition and Session was significant, *F*(1,29) = 11.382, *p* = 0.002, *η*^2^ = 0.022; this interaction reflected that participants tended to overestimate the size of their body in the psilocybin microdosing condition in the second compared to the first block, although the post-hoc tests were not significant (see Fig. 2). In line with H4_awe_ we found a significant interaction between Video, Condition, and Block Order, *F*(2,56) = 4.805, *p* = 0.012, *η*^2^ = 0.010. In contrast to our predictions however, this interaction reflected that participants tended to overestimate the size of their body in the psilocybin compared to the placebo condition to neutral videos in the first block, but not in the second block (see Fig. 2). Including absorption as covariate, as specified in H5_awe_ did not show the expected main effect (*F*(1,28) = 2.190, *p* = 0.150) and moderation effect of absorption on body size perception (*F*(2,56) = 0.030*, p* = 0.970).

**Table 3:** Body size estimation in the different experimental conditions

Video	Condition	Session	Mean	SD
Awe	Placebo	Session1	62.483	18.021
		Session2	53.95	22.569
	Psilocybin	Session1	57.9	24.82
		Session2	59.25	23.7
Ntr	Placebo	Session1	63.567	18.216
		Session2	53.25	21.348
	Psilocybin	Session1	61.6	18.367
		Session2	58.267	19.213
Pos	Placebo	Session1	61.933	20.71
		Session2	52.517	16.39
	Psilocybin	Session1	61.683	18.598
		Session2	58.733	19.158

#### Effects of psilocybin on positive aesthetic experiences

**Fig. 3:**
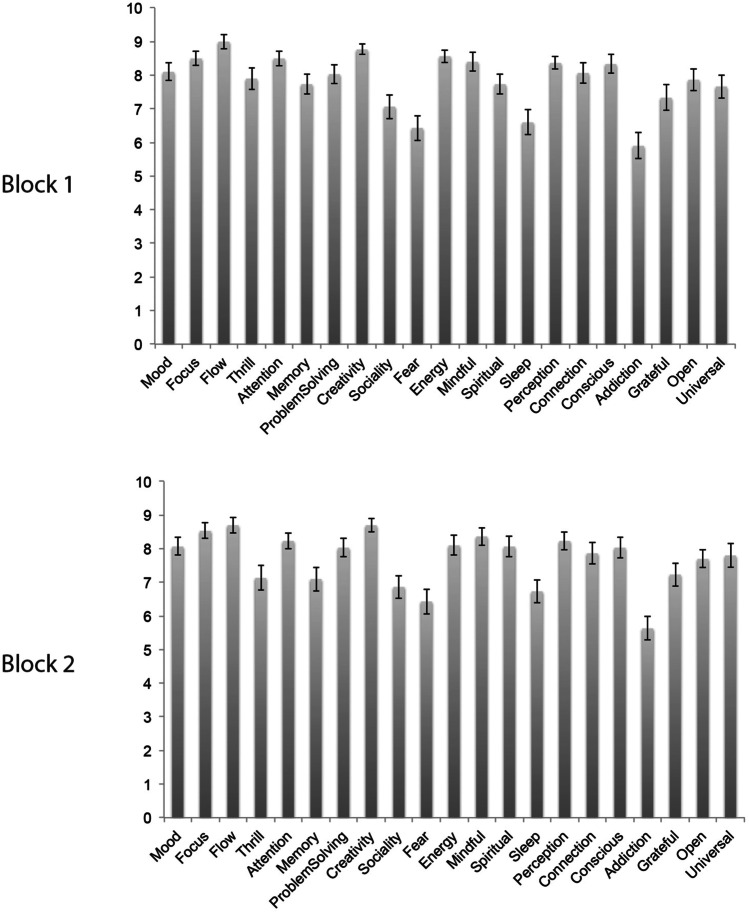
Expectations about the effects of microdosing prior to block 1 and block 2. Expectations were rated on a 10-point scale ranging from 1 = not at all to 10 = very much

The descriptives for positive aesthetic experiences in the different experimental conditions are presented in Table 4. In contrast to H1_art_, psilocybin microdosing did not affect positive aesthetic experiences (*F*(1,27) = 2.542, *p* = 0.122). In contrast to H2_art_ we also did not observe an interaction between Condition and Session (*F*(1,27) = 3.467, *p* = 0.074), nor between Condition and Block Order (*F*(1, 26) = 0.15, *p* = 0.702). Including Absorption as a covariate in the analysis revealed the hypothesized (H3_art_) main effect of Absorption, *F*(1, 26) = 7.651, *p* = 0.010: high absorption participants experienced more positive emotions to artworks. However, in contrast to the other sub-hypothesis of H3_art_, Absorption did not interact with our psilocybin manipulation (*F*(1,26) = 0.063, *p* = 0.804). We found a main effect of painter, *F*(3, 81) = 15.011, *p* < 0.001, *η*^2^ = 0.357, indicating that participants felt strongest positive aesthetic feelings in response to paintings by Kandinsky and least strong feelings in response to works by de Kooning (see Fig. 3). No other significant effects were observed.

**Table 4:** Positive aesthetic experiences in the different experimental conditions

Condition	Session	Painter	Mean	SD
Placebo	Session1	DK	22.329	20.45
		H	33.557	23.037
		K	44.471	25.736
		P	29.464	21.278
	Session2	DK	29.393	22.859
		H	36.079	24.657
		K	47.407	22.485
		P	34.371	24.545
Psilocybin	Session1	DK	25.65	23.314
		H	37.186	17.722
		K	48.8	20.542
		P	44.9	23.962
	Session2	DK	29.564	22.945
		H	35.457	24.411
		K	43.164	26.945
		P	39.264	23.636

#### Effects of psilocybin on negative aesthetic experiences

The descriptives for negative aesthetic experiences in the different experimental conditions are presented in Table 5. In contrast to H1_art_ no main effect of Condition was found, *F*(1,27) = 2.337, *p* = 0.138. H2_art_ could also not be confirmed: the interaction between Condition and Session was not significant, *F*(1,27) = 0.016, *p* = 0.899, and neither was the interaction between Condition and Block Order, *F*(1,26) = 3.662, *p* = 0.067. Finally, in line with H3_art_ we found a main effect of Absorption, *F*(1,26) = 7.915, *p* = 0.009, reflecting that high absorption participants also experienced more negative aesthetic emotions in response to artworks.

**Table 5:** Negative aesthetic experiences in the different experimental conditions

Condition	Session	Painter	Mean	SD
Placebo	Session1	DK	28	20.019
		H	22.632	18.167
		K	22.464	18.444
		P	26.75	20.013
	Session2	DK	25.027	19.648
		H	23.125	18.015
		K	23.232	18.51
		P	29.813	21.05
Psilocybin	Session1	DK	32.107	20.997
		H	31.641	18.945
		K	22.625	16.198
		P	24.304	19.708
	Session2	DK	28.563	20.456
		H	25.938	19.725
		K	25.188	21.921
		P	30.741	19.93

### Exploratory analyses

#### Drug identifications

Following each block participants were asked to guess whether they had been assigned to the microdosing or the placebo condition in the preceding weeks. They could respond by indicating “yes,” “no,” or “maybe” in response to the question “In the past few weeks, do you think you were taking an active microdose?.” A Chi^2^ analysis of the contingency table indicated that participants were breaking blind, both following the first block, Chi^2^(2) = 10.90, *p* = 0.004 (i.e., 20 out of 30 correctly guessed their condition), and following the second block, Chi^2^(2) = 13.93, *p* < 0.001 (23 out of 30 correctly guessed their condition; see Table 6).

**Table 6:** Participants guessing their condition following block 1 (upper part) and block 2 (lower part). “Yes” reflects participants guessing they were in the psilocybin microdosing condition; “no” reflects participants guessing they were in the placebo condition

	Block order		Total
	Psilo 1st	Placebo 1st	
Guess block 1			
Yes	8 (27%)	1 (3%)	9 (30%)
No	4 (13%)	12 (40%)	16 (53%)
Maybe	1 (3%)	4 (13%)	5 (17%)
Total	13 (43%)	17 (57%)	30
Guess block 2			
Yes	1 (3%)	12 (40%)	13 (43%)
No	11 (37%)	3 (3%)	14 (47%)
Maybe	1 (3%)	2 (7%)	3 (10%)
Total	13 (43%)	17 (57%)	30

#### Effect of expectations

In order to investigate to what extent participants’ prior expectations could affect the effects we observed of psilocybin microdosing on feelings of awe, we included expectations as measured prior to blocks 1 and 2 as an additional covariate in the analysis. We thus conducted a similar repeated measures ANOVA as described above, with a Condition (psilocybin vs. placebo) ∗ Session (Session1 vs. Session2) ∗ Video (awe, positive, neutral) design, with “expectations” as an additional covariate.

When prior expectations in block 1 were included as covariate, the main effect of Condition was no longer significant (*F* = 0.086, *p* = 0.772). A main effect of expectations, *F*(1,28) = 4.80, *p* = 0.037, *η*^2^ = 0.146, indicated that participants with stronger expectations experienced more profound feelings of awe. The same pattern emerged when including expectations prior to the second block as covariate, also rendering the effect of Condition mute (*F* = 0.266, *p* = 0.610), while the main effect of expectations was significant, *F*(1,28) = 9.210, *p* = 0.005, *η*^2^ = 0.248.

In a post-hoc analysis we decided to provide a more in-depth analysis of the expectations that people reported. Participants’ expectations prior to each block are represented in Fig. 3. As can be seen, the expectations were comparable prior to both blocks; only the expectation to experience more thrill was lower following the first block, *t*(29) = 3.22, *p* = 0.003. The strongest expectations regarding microdosing were to experience increased flow and creativity, while participants had lower expectations regarding the effects on fear, sleep, and addiction.

## Discussion

In this study we set out to investigate the effects of psilocybin microdosing on feelings of awe and aesthetic emotions. Psilocybin microdosing increased feelings of awe—mostly in response to the positive and neutral control videos. The positive and neutral control videos mainly presented funny animals, manmade vehicles, and boring landscapes. Microdosing may have helped participants to experience more awe in response to the content of these videos. We found that the effect of microdosing on awe was most pronounced in the first compared to the second session. This could reflect that over time participants habituated to psilocybin and its psychological effects. We did not observe effects of psilocybin microdosing on positive or negative emotions in response to the viewing of abstract artworks.

Despite the use of a double-blind placebo-controlled design, most participants broke blind and correctly guessed their condition, even though we kept the dosages low at less than 1/10th of a full psychedelic dose. Anecdotally, during the lab sessions participants indicated that they could infer their condition based on subtle side effects, i.e., bodily signals, such as increased sweating, heart rate, or a dry mouth. This mirrors findings from clinical trials with anti-depressants, indicating that approximately 80% of the patients correctly guess their condition based on physiological side effects (Rabkin et al. 1986). A potential remedy against the breaking-blind problem would be the use of even lower levels of psilocybin microdoses as compared to the present study, or the inclusion of an active placebo condition, such as niacin. The breaking blind problem could partially underlie the effects that we observed of microdosing on feelings of awe. Participants may have engaged in a (implicit) process of motivated reasoning and socially desirable responding, by over-reporting their feelings of awe in the psilocybin compared to the placebo condition. Alternatively, our findings could also reflect a misattribution of arousal (Sinclair et al. 1994), especially because the awe questions that we used were quite generic and may have captured both valence and arousal of the emotion (see also “Constraints on generality” section). That is, the psilocybin microdosing condition may have induced a more arousing state, which subsequently affected participants’ perceived emotions. A remedy for this potential concern is to use active placebos and/or to systematically assess the dose–response relationship at different levels of microdoses (Hutten et al. 2020). However, we did not observe a consistent effect of microdosing on art perception, which argues against the idea that the effect of microdosing on awe reflects a general expectancy-effect or an effect of arousal on emotion.

We found that the personality trait of absorption was positively related to both feelings of awe and to art perception: high absorption participants overall reported stronger feelings of awe and both more positive and negative aesthetic emotions in response to the artworks. These findings are in line with earlier studies (van Elk et al. 2016; Wild et al. 1995) and align with the notion that absorption captures an experiential mindset that intensifies both inner and outer sensory experience (Lifshitz et al. 2019). In our pre-registration we also specified a hypothesis about a moderation effect such that the effects of microdosing on felt awe would be most pronounced for high absorption participants (https://osf.io/cn8z4/). Our analyses did not confirm this effect, but we note that due to participant dropout our study may have been underpowered to detect a moderation effect.

Overall, participants had quite strong expectations regarding the alleged benefits of microdosing, which may be related to the microdosing workshop, which could have further boosted participants’ expectations. Expectations remained high throughout the study: only thrill seeking decreased after the first block, likely because participants were less excited than when the study started. Participants’ expectations about microdosing might mirror their own experiences with psychedelics—as many of our participants had prior experience with using psychedelics. These expectations in turn may have made them more likely to feel and report awe in response to videos that are not very conducive in and of themselves.

## Constraints on generality

Given previous concerns about the replicability of psychedelic research and of microdosing studies in particular (Petranker et al. 2020a, b), we hereby explicitly acknowledge the potential limits and the constraints on generalizability of this study.

We note that the items that we used in the present study to measure feelings of awe may have a high face validity, but that we did not provide a full psychometric validation of the items that we included. Specifically, our items primarily capture the self-transcendent nature of feelings of awe, whereas for instance the need to revise one’s mental schemes is not covered by the questions that we used. Future studies on awe should preferably use well-validated measures and instruments to measure feelings of awe, such as the Awe Experience Scale (Yaden et al. 2019).

A main limitation of the present study is that there was a strong selection bias, as this study was set up through a microdosing workshop, in which participants voluntarily participated without any financial remuneration. Informal observation indicated that most participants had prior experience with psychedelics, which might explain the high number of participants breaking blind to their experimental condition. We also had a high attrition rate, which might be well related to some participants becoming disappointed or frustrated with the effects of psilocybin microdosing—or simply because of the fact that our testing schedule was quite intense and required a high degree of commitment. The participants who actually remained in the study, may have been extremely motivated to contribute to the science of microdosing, as they voluntarily subjected themselves to an extensive testing battery for four times, while also completing several questionnaires in between.

The selection bias and motivation of participants may explain the expectancy-effects that we observed in our study. We also note that the workshop provided to the participants was a unique event that took place in quite a specific setting (i.e., the basement of a Smartshop in the center of Amsterdam). As this study was set up as a combined field- and lab-based study the generalizability and replicability may prove to be difficult. For this, more systematic and controlled lab-based studies are required. However, if anything, our study setup may have optimally boosted participants’ responses to the psilocybin microdosing—but even under these circumstances, the effects of microdosing appear to be rather small or even non-existent.

## Conclusions

Psilocybin microdosing enhanced feelings of awe in response to videos of funny animals and moving objects, though the effects were likely driven by participants’ prior expectations and participants were breaking blind. Future studies on microdosing should therefore take expectancy-effects into account in the study design and it is recommended to use active placebos as a remedy to the breaking-blind problem. Finally, the inclusion of more ecologically valid measures including awe and art perception, promising avenues to capture eventual effects of perceptual and sub-perceptual doses of psychedelics.

### Supplementary Information

Below is the link to the electronic supplementary material.

Supplementary file1 (DOCX 15 KB)

## Appendix. Microdosing expectations questionnaire

Participants indicated to what extent the statement applied to them by using a 10-point scale, ranging from 1 = not at all to 10 = very much.

In general: what are your expectations from practicing microdosing? Are there any specific benefits that you hope to obtain from microdosing?

By microdosing I hope to….

Improve my mood.

Increase my focus.

Get more easily into the flow.

Get a thrill out of daily life.

Increase my attention.

Improve my memory.

Improve my problem solving skills.

Become more creative.

Feel more comfortable in social situations.

Reduce my fear/phobias.

Feel more energized.

Become more mindful.

Feel more spiritual.

Improve my sleep.

Enhance my perception.

Feel more connected to others.

Be more conscious in daily life.

Quit smoking or other addictions.

Become more grateful.

Become more open.

Feel more connected to the universe.
