# Supplementary material for: Effects of psilocybin microdosing on awe and aesthetic experiences: a preregistered field and lab-based study
Source: Psychopharmacology (Berl). 2021 Apr 30;239(6):1705–20. doi: 10.1007/s00213-021-05857-0 (PMC9166866; doi:10.1007/s00213-021-05857-0)
Supplement: Supplementary file 1 — Supplementary file1 (DOCX 15 KB) [file 213_2021_5857_MOESM1_ESM.docx]

**Supplementary Material Online**

*Drugs and chemicals*

Analytical standards of psilocin, psilocybine, norbaeocystine, and baeocystine were synthesized in-house (purity ≥95%) in the Department of Organic Chemistry, University of Chemistry and Technology Prague (UCT). Methanol and formic acid were of LC-MS grade and were purchased from Merck (Czech Republic). Ultra pure water, 18.2 MΩ-cm, was produced using a Smart2Pure 12 water purification system (Thermo Scientific, Germany). The stock solutions of psilocin and psilocybine were prepared in methanol and the stock solutions of norbaeocystine and baeocystine in 50% (*v/v*) methanol (at a concentration 1 mg/mL and stored at -20 °C). A working solution contained a mixture of analytes was diluted with methanol to 10 µg/mL.

*Sample preparation*

Capsules were opened and 100 mg of inner solid was weighted to dark glass tubes with screw caps. Samples were dissolved in 5 mL of methanol pre-purged with nitrogen gas. A rack with samples was covered with aluminium foil and vortexed for 150 min. Mixtures were centrifuged for 15 min at 2,000 rpm (25°C). 100 µL aliquot was diluted with 900 µL of 0.1% (*v/v*) formic acid. Samples were 100 times diluted with 0.1% (*v/v*) formic acid because of high concentrations of psilocin and psilocybine. 5 µL was injected to the LC-MS and samples were prepared at triplicate.

*LC conditions*

The LC system used was Agilent 1290 Infinity (Agilent Technologies, USA) and conditions were as follows: a column Poroshell 120 Phe Hex, 2.1 × 100 mm, 2.7 µm, with pre-column, gradient elution with 0.1% (*v/v*) formic acid (mobile phase A) and methanol containing 0.1% (*v/v*) formic acid (mobile phase B) with a flow rate of 300 µL/min. The injection volume was 3 µL with 3 s needle wash in a flush port. The gradient setup was: 0-3.5 min from 90%A to 65%A, 3.5-4.0 min from 65%A to 0%, till 4.5 min held on 0%A, 4.5-5.0 min back to 90%A and equilibrated at 90%A to 8 min. The valve arrangement: 1-4 min to the MS source.

*MS conditions*

The tandem MS used was 6460 Triple Quad LC/MS (Agilent Technologies, USA) with Jet Stream Electrospray Ionisation Source. Dynamic multiple reaction monitoring (dMRM) method was used. Briefly the conditions of the instrument were as follows: positive ion mode, ionisation voltage of 2300 V, source temperature of 340°C and gas flow rate 10 L/min, values for sheath gas were 400°C and 12 L/min, and for nebulizer 30 psi. Data was acquired and evaluated with MassHunter software (Agilent Technologies, USA).

**Supplementary Table 1** LOD was calculated as 3×S/N (signal to noise ratio) and LOQ as 10×S/N (Note: The left part of the table shows the final concentrations measured owing to LC-MS; the right side of the table shows the calculation on 1 g of capsule inner solid).

|  | *ng/ml* | | *µg/g (ppm)* | |
| --- | --- | --- | --- | --- |
|  | **LOD** | **LOQ** | **LOD** | **LOQ** |
| **norbaeocystin** | 5.0 | 10.0 | 2.5 | 5.0 |
| **baeocystin** | 1.0 | 5.0 | 0.5 | 2.5 |
| **psilocybin** | 0.5 | 1.0 | 0.3 | 0.5 |
| **psilocin** | 1.0 | 5.0 | 0.5 | 2.5 |
